# TWEAK/Fn14 Signalling Regulates the Tissue Microenvironment in Chronic Pancreatitis

**DOI:** 10.3390/cancers15061807

**Published:** 2023-03-16

**Authors:** N. Dianah B. Abu Bakar, Rodrigo Carlessi, Jully Gogoi-Tiwari, Julia Köhn-Gaone, Vincent Williams, Marco Falasca, John K. Olynyk, Grant A. Ramm, Janina E. E. Tirnitz-Parker

**Affiliations:** 1 Curtin Medical School, Curtin Health Innovation Research Institute, Curtin University, Bentley, WA 6102, Australia; 2 Fiona Stanley Hospital, Murdoch, WA 6150, Australia; 3 School of Medical and Health Sciences, Edith Cowan University, Joondalup, WA 6027, Australia; 4 QIMR Berghofer Institute, Brisbane, QLD 4006, Australia; 5 Faculty of Medicine, The University of Queensland, Brisbane, QLD 4006, Australia; 6 Harry Perkins Institute of Medical Research and Centre for Medical Research, The University of Western Australia, Nedlands, WA 6009, Australia

**Keywords:** choline-deficient, ethionine-supplemented diet, TWEAK/Fn14 signalling, chronic pancreatitis, ductular proliferation, inflammation, fibrosis

## Abstract

**Simple Summary:**

The TWEAK/Fn14 signalling pathway has emerged as a major regulator of tissue injury and regeneration through its pro-proliferative, pro-inflammatory, and pro-fibrotic cellular effects. Its role in pancreatic injury and cancer has not been elucidated. In this study, we confirmed the choline-deficient, ethionine-supplemented diet as a suitable murine model to study chronic pancreatitis and demonstrated that TWEAK/Fn14 signalling plays a significant role in the establishment and progression of the chronic pancreatitis tissue microenvironment.

**Abstract:**

Chronic pancreatitis increases the risk of developing pancreatic cancer through the upregulation of pathways favouring proliferation, fibrosis, and sustained inflammation. We established in previous studies that the ligand tumour necrosis factor (TNF)-like weak inducer of apoptosis (TWEAK) signals through its cognate receptor fibroblast growth factor-inducible 14 (Fn14) to regulate these underlying cellular processes in the chronic liver injury niche. However, the role of the TWEAK/Fn14 signalling pathway in pancreatic disease is entirely unknown. An analysis of publicly available datasets demonstrated that the TWEAK receptor Fn14 is upregulated in pancreatitis and pancreatic adenocarcinoma, with single cell RNA sequencing revealing pancreatic ductal cells as the main Fn14 producers. We then used choline-deficient, ethionine-supplemented (CDE) diet feeding of wildtype C57BL/6J and Fn14 knockout littermates to (a) confirm CDE treatment as a suitable model of chronic pancreatitis and (b) to investigate the role of the TWEAK/Fn14 signalling pathway in pancreatic ductal proliferation, as well as fibrotic and inflammatory cell dynamics. Our time course data obtained at three days, three months, and six months of CDE treatment reveal that a lack of TWEAK/Fn14 signalling significantly inhibits the establishment and progression of the tissue microenvironment in CDE-induced chronic pancreatitis, thus proposing the TWEAK/Fn14 pathway as a novel therapeutic target.

## 1. Introduction

Chronic pancreatitis is the long-term necroinflammation of the pancreas, which is associated with enzymatic tissue autodigestion and significantly impaired pancreatic exocrine function. It often develops on the background of excessive alcohol consumption, tobacco smoking, and obesity, and may result from recurrent episodes of acute pancreatitis [1]. Chronic pancreatitis patients have a nearly eight-fold increased risk of developing pancreatic cancer five years post-initial diagnosis [2]. This is not surprising, considering that the molecular and cellular microenvironment is significantly altered towards a potentially pro-tumorigenic phenotype in chronic pancreatitis, driven by acinar atrophy and sustained inflammation, tissue scaring or fibrosis, and chronic ductal proliferation. Eventually, these interlinked cellular processes may lead to low- and high-grade dysplastic pancreatic intraepithelial neoplasia, and in the most progressed stages to invasive adenocarcinoma [3]. Historically, there has been a lack of animal models that faithfully mimic the human disease and the choice of an appropriate model depends on the underlying research question. One of the best characterised rodent models of pancreatitis involves repeated intravenous or intraperitoneal injections of caerulein, a cholecystokinin peptide orthologue that induces pancreatic enzyme activation, acinar cell injury, and infiltration of inflammatory cells in response to digestive necrosis. However, the pathobiology in caerulein-induced pancreatitis is rather mild and is more suited to study the initial mechanisms of acute rather than chronic pancreatitis [4]. In early studies, feeding female mice a choline-deficient, 0.5% ethionine-supplemented (CDE) diet was used to induce acute and severe pancreatic injury. Male mice only showed signs of acute CDE-induced pancreatitis when pre-treated with oestrogen. Importantly, mortality rates reached 100% in female mice only four days after CDE induction when 0.5% ethionine was used, making it necessary to cycle the animals through periods of CDE treatment, followed by a recovery period (all CDE-induced pancreatitis studies reviewed in [5]). Our laboratory uses feeding of a choline-deficient diet and the administration of 0.15% ethionine in the drinking water to induce chronic liver injury in mice [6], which is characterised by epithelial cell death as well as progressive inflammation, ductular proliferation, and fibrosis [7]. One signalling pathway that was identified to be a major regulator of these underlying processes is induced by tumour necrosis factor (TNF)-like weak inducer of apoptosis (TWEAK) binding to its cognate receptor fibroblast growth factor-inducible 14 (Fn14), with the ligand and receptor being expressed by TNFSF12 and TNFRSF12A, respectively. TWEAK/Fn14 signalling drives regeneration in early CDE-induced injury [8], co-regulates liver progenitor cell and fibrogenic responses [9], and promotes the establishment and maintenance of the liver tumour microenvironment [10]. This current study aimed to characterise the CDE diet as a model of chronic pancreatitis in male mice using 0.15% ethionine and to establish via genetic ablation whether the TWEAK/Fn14 pathway has any significance in chronic pancreatic injury, which is characterised by pathological responses similar to hepatic disease. Our data confirm long-term CDE treatment as an experimental regime that recapitulates the hallmarks of chronic pancreatitis and will initiate further studies evaluating TWEAK/Fn14 signalling as a novel therapeutic target in pancreatic disease.

## 2. Materials and Methods

### 2.1. Animal Models

Fn14 knockout (KO) mice [11] (kindly provided by Biogen, Cambridge, MA, USA) and their inbred wildtype littermates (colonies maintained and bred at the Animal Resources Centre, Murdoch, WA, Australia) were housed on wheaten chaff bedding and kept on 12-h day/night cycles in individually ventilated cages. At six weeks of age, randomly grouped mice were given *ad libitum* access to either a normal chow and drinking water or a choline-deficient diet (MP Biomedicals, Seven Hills, NSW, Australia) and water that contained 0.15% DL-ethionine (Sigma-Aldrich, Castle Hill, NSW, Australia), as previously described [12]. Pancreas tissues were harvested three days, three months and six months of CDE treatment. All animal experiments were performed in accordance with the Australian code for the care and use of animals for scientific purposes at Curtin University, Perth, Australia, with local animal ethics committee approval (AEC_2014_28).

### 2.2. Histology, Immunohistochemistry, and Immunofluorescence Experiments

The pancreas tissue was divided into equal parts for (a) formalin-fixation and paraffin-embedding (FFPE) for histology and immunohistochemistry experiments, or (b) cryopreservation through embedding in Tissue-Tek OCT compound and snap-freezing for immunofluorescent staining procedures. FFPE tissue was sectioned at 4 µm, dewaxed, and rehydrated according to standard protocols. Haematoxylin and eosin (Dako, Agilent Technologies Australia, Mulgrave, VIC, Australia) as well as Picrosirius Red (PSR; Polysciences Inc., Warrington, PA, USA) histochemical stainings were performed according to the manufacturers’ instructions. Positively PSR-stained areas were quantified using Image J 1.51k software (https://imageJ.nih.gov, accessed on 15 October 2022). The pancreatic tissues were assessed for histopathological changes and features of chronic pancreatitis by an experienced histologist (V. Williams). Snap-frozen tissue sections, cut at 7 µm thickness, were either fixed in ice-cold methanol/acetone (1:1, VWR, Brisbane, QLD, Australia) and air-dried, or fixed in 4% paraformaldehyde (Sigma-Aldrich, Castle Hill, NSW, Australia) and permeabilised with 0.2% Triton-X-100 (Amresco, Cleveland, OH, USA). Sections were then rehydrated in phosphate-buffered saline (PBS) before incubation in serum-free protein block (Dako, North Sydney, NSW, Australia) and antibody labelling. The primary antibodies of rabbit anti-amylase (1:300; Abcam, Melbourne, VIC, Australia), rabbit anti-cytokeratin (CK) 7 (1:4000, Abcam, Melbourne, VIC, Australia), rat anti-CK19 (1:200; TROMA-III, Developmental Studies Hybridoma Bank, Iowa City, IA, USA), mouse α-smooth muscle actin (1:500, Sigma-Aldrich, Castle Hill, NSW, Australia), rat anti-CD45 (1:200; eBioscience, San Diego, CA, USA), rat anti-F4/80 (1:150, eBioscience), rat anti-CD11b (1:400, eBioscience, San Diego, CA, USA), and rat anti-Ly6G (1:50, eBioscience, San Diego, CA, USA) were diluted in antibody diluent (Dako, North Sydney, NSW, Australia), detected with Alexa Fluor-labelled secondary antibodies (1:200; Life Technologies, Scoresby, VIC, Australia) and visualised using an Olympus BX51 or Olympus IX51 fluorescent microscope (Olympus Corporation, Tokyo, Japan). The nuclei were counterstained with 4′-6-diamidino-2-phenylindole (DAPI; Molecular Probes, Eugene, OR, USA). Cell quantification was performed through manual cell counting of non-overlapping fields of view and using the automated Zeiss Axio Scan.Z1 Digital Slide Scanning System.

### 2.3. Triple Fluorescent Staining of Pancreatic Islet Cells

FFPE pancreas sections of 4 µm thickness were dewaxed and rehydrated according to standard protocols. The sections were immersed in a sodium citrate buffer (10 mM sodium citrate, pH 6.0) and subjected to heat-induced epitope retrieval. After rehydration in PBS, the sections were treated with a serum-free protein block (Dako, North Sydney, NSW, Australia) for 20 min, followed by primary antibody incubations at 4 °C overnight using guinea pig anti-insulin (1:200, Abcam, Melbourne, VIC, Australia), mouse anti-glucagon (1:200, Abcam, Melbourne, VIC, Australia), and rabbit anti-somatostatin (1:250, Abcam, Melbourne, VIC, Australia), all diluted in antibody diluent (Dako, North Sydney, NSW, Australia). Visualisation was performed using Alexa Fluor dyes (Life Technologies, Scoresby, VIC, Australia) and was imaged using a confocal Nikon A1+ point scanning confocal microscope.

### 2.4. TNFRSF12A Gene Expression Analysis

Gene expression analysis of TNFRSF12A in human pancreatic adenocarcinoma compared to normal pancreas tissue was conducted on data extracted from the TCGA PAAD and GTEx datasets using the web server for expression profiling GEPIA2 [13]. Log_2_FC cut-off was set to 2 and the *p*-value cut-off set to 0.0001 by one-way ANOVA. For Kaplan–Meier survival analysis, patients were first ranked according to the expression values of TNFRSF12A into high (top quartile) or low (bottom quartile), then overall survival was plotted and 95% confidence intervals, hazard ratio, and p values were calculated using the log-rank test.

Differential gene expression analysis of mouse Tnfrsf12a in healthy versus pancreatitis was conducted using data from three independent publicly available bulk RNA-seq datasets of caerulein-induced pancreatitis. Raw or normalised counts were obtained from the Gene Expression Omnibus (GEO) repository. For GSE119844, a normalised count matrix in transcripts per million (TPM) was directly downloaded from the database, and TPM values for Tnfrsf12a expression were directly compared between control and caerulein-treated mice. For GSE115758 and GSE169525, raw counts were downloaded, then downstream normalisation to counts per million reads (CPM) and differential expression analysis were performed using Edger [14] in R.

Single cell RNA sequencing data of the human pancreas was obtained from GSE81547 [15] and visualisations were created using the UCSC Cell Browser [16].

### 2.5. Statistical Analysis

Statistical significance was assessed with one-way analysis of variance (ANOVA) and Tukey’s post-test using GraphPad Prism and InStat 3 for Macintosh (GraphPad Software Inc., San Diego, CA, USA). Data were expressed as mean ± standard error of the mean. Statistical significance was expressed as * *p* < 0.05, ** *p* < 0.01, *** *p* < 0.001 within the same genetic background (experimental group compared to healthy controls) and ^#^ *p* < 0.05, ^##^ *p* < 0.01, ^###^ *p* < 0.001 when data were significantly different between the CDE-fed wildtype (WT) and knockout (KO) animals at the same time point.

## 3. Results

### 3.1. The Fn14 Receptor Is Upregulated in Chronic Pancreatitis and Pancreatic Adenocarcinoma

**Figure 1:**
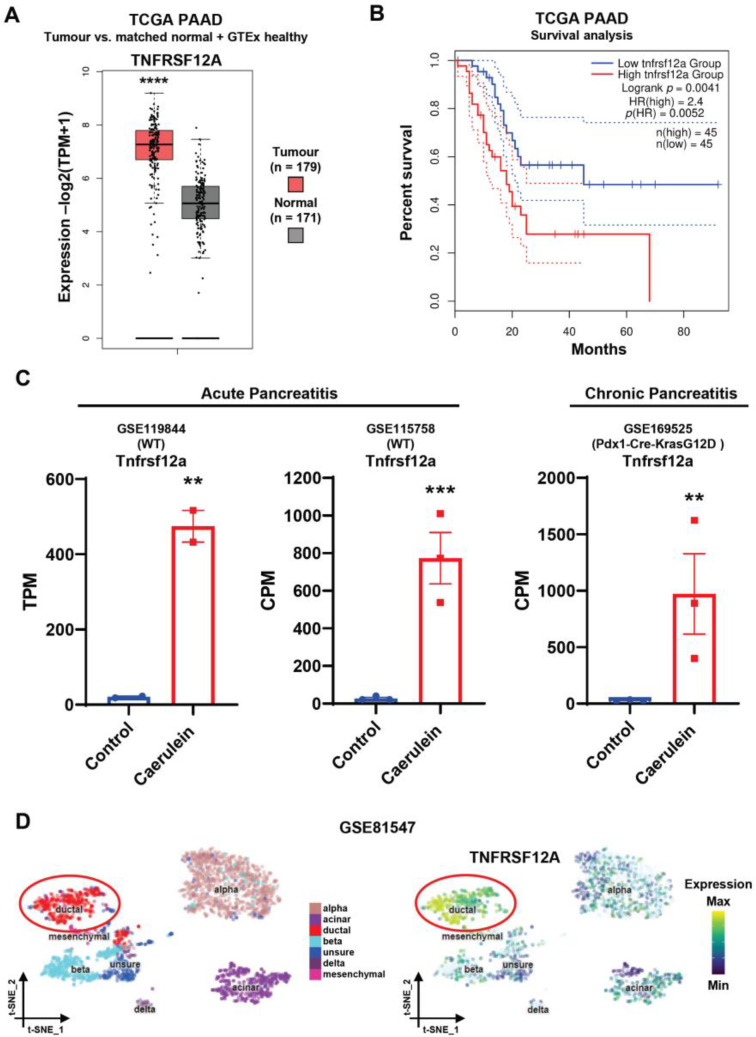
The TWEAK receptor Fn14 is upregulated in pancreatic adenocarcinoma, as well as acute and chronic pancreatitis. Box plots depicting the expression levels of human TNFRSF12A in pancreatic adenocarcinoma tumour samples from the TCGA PAAD dataset (tumour, n = 179) in comparison with adjacent normal tissue and healthy human pancreas from the GTEx dataset (normal, n = 171). Log2FC cut-off = 2, **** *p* < 0.0001 by one-way ANOVA (**A**) Kaplan–Meier survival analysis of TCGA-PAAD patients ranked high (top quartile, n = 45) and low (bottom quartile, n = 45) in terms of their expression levels of TNFRSF12A. Hazard ratio (HR) and *p* values were calculated by the log-rank test; 95% confidence intervals are denoted by the dotted curves. (**B**) Expression levels of mouse Tnfrsf12a in three independent publicly available bulk RNA-Seq datasets of Caerulein-induced pancreatitis. GSE119844 and GSE115758 correspond to acute, and GSE169525 chronic exposure to Caerulein. TPM, transcripts per million; CPM, counts per million reads. Data are mean ± SEM, ** *p* < 0.01, *** *p* < 0.001 by unpaired *t*-test (**C**) (**Left**) t-distributed stochastic neighbour embedding (t-SNE) visualisation of scRNA-Seq data (GSE81547) on 2544 human pancreatic cells. Seven clusters are annotated and identified as alpha, beta, delta, mesenchymal, acinar, and ductal cells. One remaining cluster corresponds to an unidentified cell population (unsure). (**Right**) Expression distribution of TNFRSF12A across the t-SNE space highlighting a higher expression by ductal cells (**D**).

Publicly available datasets from The Cancer Genome Atlas (TCGA Research Network, https//www.cancer.gov/tcga, accessed on 1 April 2022) demonstrated that TNFRSF12A is strongly expressed in human pancreatic adenocarcinoma (PAAD) patients (n = 179), with a significantly lower expression in patient-matched non-tumorous pancreas tissue (n = 171), suggesting that Fn14 plays a role in PAAD biology (Figure 1A). Importantly, a high expression of TNFRSF12A is associated with more aggressive PAAD, illustrated by the significantly shorter patient survival compared with the low TNFRSF12A expression group (Figure 1B; n(high) = 45, n(low) = 45, Logrank *p* = 0.0041). As PAAD may be a sequela of pancreatitis, we further interrogated publicly accessible data of the murine caerulein model (https://ncbi.nlm.nih.gov, accessed on 1 April 2022; datasets GSE119844 for acute pancreatitis and GSE169525 for chronic pancreatitis) and found that TNFRSF12A expression was low in healthy pancreas tissue and highly upregulated in acute and chronic caerulein-induced pancreatitis (Figure 1C). Furthermore, single cell RNA sequencing of human non-diabetic pancreas donors with BMI < 30 [15] revealed that pancreatic ductal cells are the main cellular source of TNFRSF12A (Figure 1D, GSE81547).

### 3.2. Long-Term Choline-Deficient, Ethionine Diet Treatment Leads to Chronic Pancreatitis

**Figure 2:**
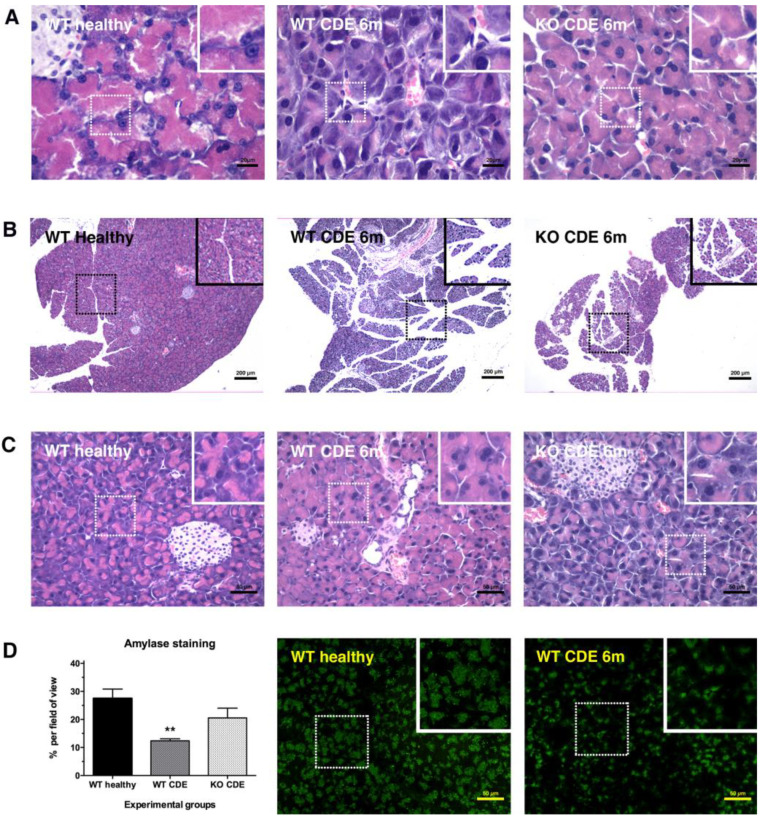
Lack of Fn14 signalling reduces the histological hallmarks of CDE-induced chronic pancreatitis. Fn14 knockout (KO) mice on a long-term CDE diet show reduced peri-acinar inflammation (**A**), dilation of inter-lobular ducts (**B**), and less pronounced reductions in zymogenic granule density than CDE-fed wildtype (WT) controls at the same 6-month time point (**C**). Pancreatic amylase expression was only significantly reduced in 6-month CDE-treated WT animals compared with healthy pancreas tissue and not in mice that lacked Fn14 signalling activity (**D**). Data are presented as means ± SEM, with statistical significance represented as ** *p* < 0.01.

To gain more insight into the significance of TWEAK/Fn14 signalling in pancreatic disease, we utilised wildtype and Fn14-deficient mice, and subjected them to pancreatic injury by feeding the mice a CDE diet. First, we had to confirm that CDE treatment resulted in histopathological changes resembling human pancreatitis. An analysis of 6-month CDE-treated wildtype pancreas tissue clearly demonstrated an inflammatory response in the peri-acinar spaces (Figure 2A), dilation of interlobular ducts (Figure 2B), significantly reduced density of zymogenic granules (Figure 2C), and significantly reduced amylase expression compared with the healthy controls (Figure 2D). A detailed blind evaluation by an experienced histologist (V. Williams) confirmed that long-term CDE feeding induced features of chronic pancreatitis and thus served as a suitable model for further investigations. Interestingly, the pancreata of 6-month CDE-treated Fn14 KO mice showed only mild signs of pancreatitis and were closer in appearance to the healthy controls than to their chronically injured wildtype counterparts, further underlining the potential relevance of TWEAK/Fn14 signalling during chronic pancreatitis (Figure 2A–D).

**Figure 3:**
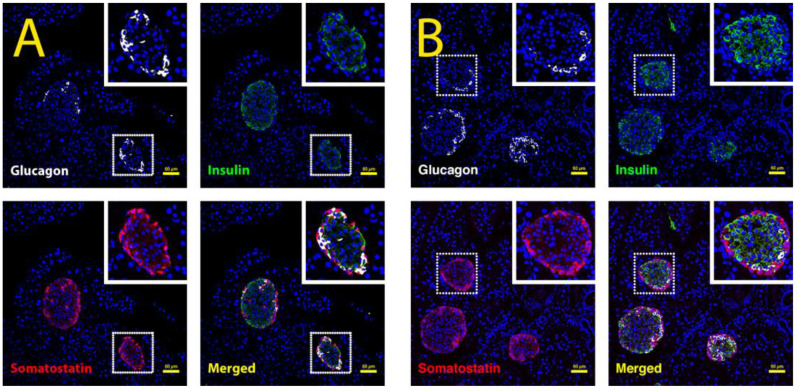
Confocal analysis of pancreatic islets in CDE-treated WT and Fn14 KO mice. Glucagon^+^ alpha cells (white), insulin^+^ beta cells (green), and somatostatin^+^ delta cells (red) in chronically injured Fn14 KO mice (**B**) are confocally compared to Fn14 WT animals (**A**) and determined not to be affected by a lack of Fn14 signalling activity.

Confocal analysis of glucagon^+^ alpha cells, insulin^+^ beta cells, and somatostatin^+^ delta cells further revealed that pancreatic islets were not altered histologically. Thus, the endocrine pancreas was not affected by long-term choline deficiency and ethionine treatment (Figure 3A,B).

### 3.3. TWEAK/Fn14 Signalling Regulates the Pancreatic Ductular Reaction

**Figure 4:**
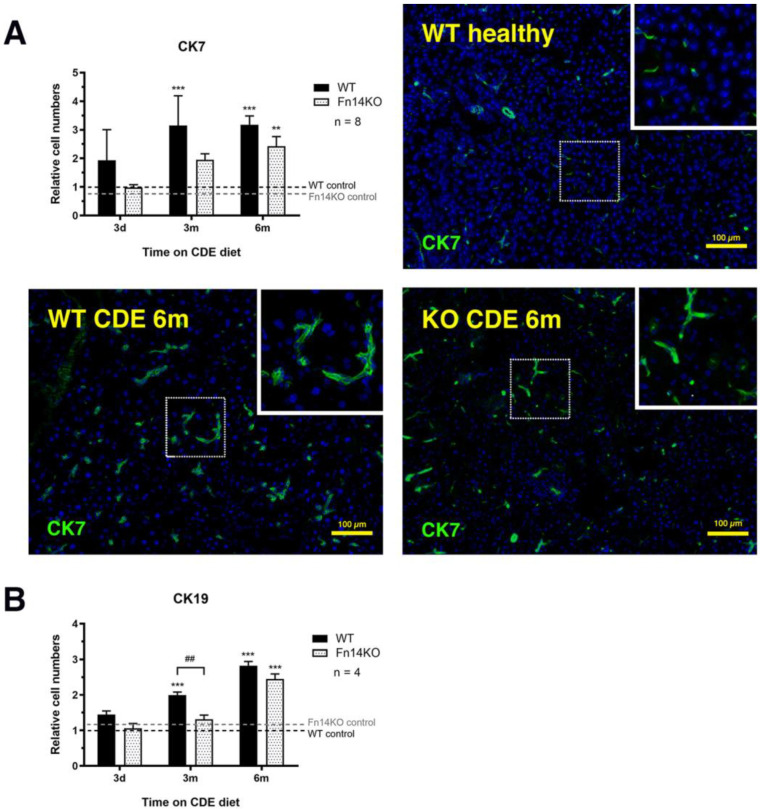
Lack of Fn14 signalling activity delays the pancreatic ductular reaction. Immunofluorescent staining and quantitation of cytokeratin (CK) 7^+^ (**A**) and CK19^+^ pancreatic ducts (**B**) (photomicrographs not shown) reveals a delay in ductular proliferation in CDE-fed Fn14 KO compared to chronically injured WT mice. (**A**) Data are presented as means ± SEM, with statistical significance represented as ** *p* < 0.01 and *** *p* < 0.001. (**B**) Data are presented as means ± SEM, with statistical significance represented as *** *p* < 0.001 comparing groups of the same genetic background and ## *p* < 0.01 comparing CDE-treated WT and Fn14 KO mice.

We next assessed the cellular compartments of the exocrine pancreas in CDE-treated tissue and compared wildtype to Fn14 knockout mice that were not able to signal through the TWEAK pathway in response to pancreatic injury. Cytokeratins 7 and 19 are expressed in healthy pancreatic ductal cells, as well as the ductal adenocarcinoma [17]. Immunofluorescent staining and quantification of CK7^+^ and CK19^+^ tubular complexes showed a significant increase in CDE-treated wildtype mice, representing the regenerative response to chronic exocrine pancreas injury, consistent with the well-established proliferation of CK7^+^/CK19^+^ hepatic ductal cells during chronic liver injury [18]. However, the cell numbers of both populations were significantly reduced in Fn14 knockout mice at the 3-month and 6-month time point (Figure 4, representative photomicrographs of CK19 in Supplementary Figure S1).

### 3.4. Fibrosis Is Reduced in CDE-Treated Fn14 Knockout Mice

**Figure 5:**
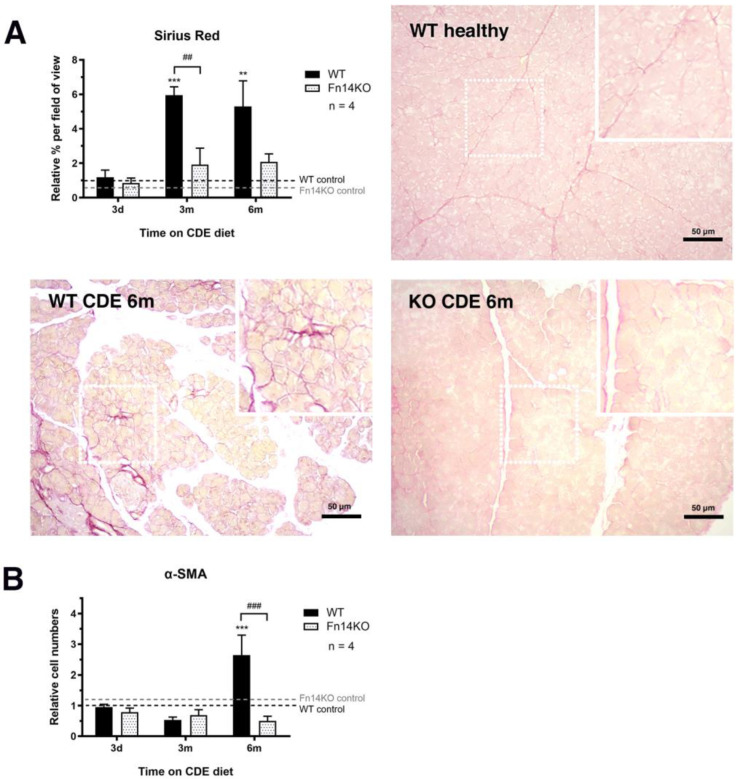
Lack of Fn14 signalling activity significantly reduces CDE-induced fibrosis development. Sirius Red staining (**A**) and immunohistochemical staining for α-smooth muscle actin (α-SMA)^+^ pancreatic stellate cells (**B**) (photomicrographs not shown) demonstrate significantly reduced fibrogenesis in CDE-fed Fn14 KO compared with chronically injured WT mice. (**A**) Data are presented as means ± SEM, with statistical significance represented as ** *p* < 0.01 and *** *p* < 0.001 comparing groups of the same genetic background and ## *p* < 0.01 comparing CDE-treated WT and Fn14 KO mice. (**B**) Data are presented as means ± SEM, with statistical significance represented as *** *p* < 0.001 comparing groups of the same genetic background and ### *p* < 0.001 comparing CDE-treated WT and Fn14 KO mice.

Both wildtype and Fn14 knockout mice showed fibrogenic changes with long-term CDE treatment, characterised by a mild thickening of the interlobular and periductular collagen, with some degree of pancreatic fibrosis in the peri-acinar spaces, as visualised by Picrosirius Red staining. However, the fibrogenic response to injury was significantly less pronounced in the Fn14 knockout mice at three and six months of CDE feeding (Figure 5A). We further investigated α-smooth muscle actin staining as a measure of fibrosis-driving pancreatic stellate cells [19]. While the numbers of α-SMA^+^ cells increased substantially over time in the wildtype mice, they remained low in animals that were not able to signal through the TWEAK/Fn14 pathway, highlighting the importance of Fn14 downstream events for pancreatic stellate cell activation during chronic injury (Figure 5B, representative photomicrographs of α-SMA in Supplementary Figure S2).

### 3.5. Pancreatic TWEAK/Fn14 Signalling Is Important for Inflammatory Cell Recruitment

**Figure 6:**
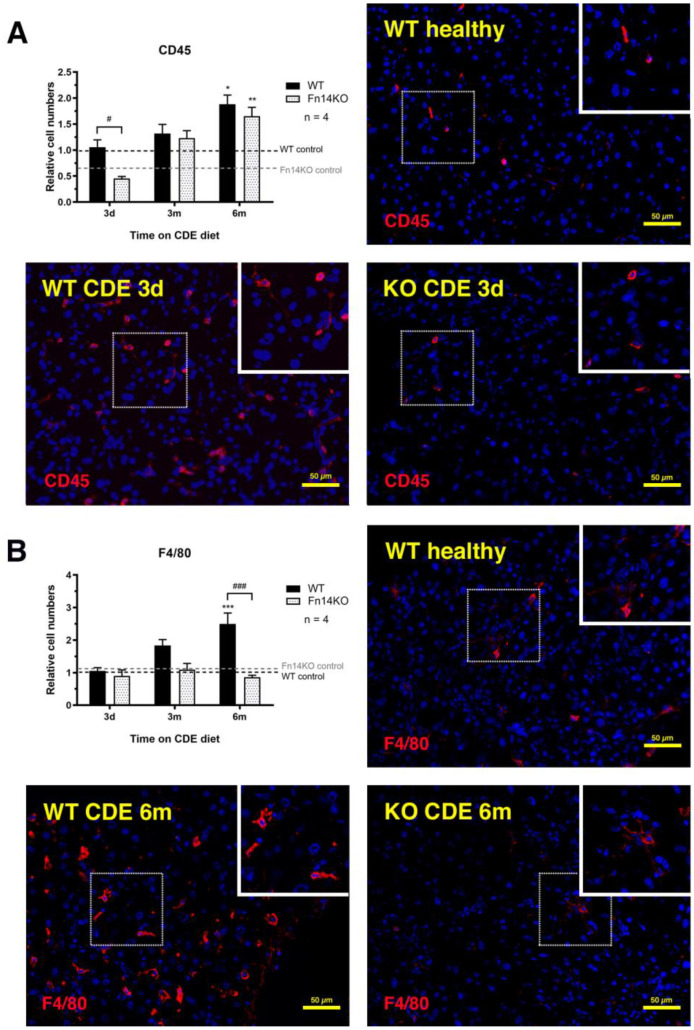
Inflammatory cell numbers are significantly reduced in pancreata of CDE-treated Fn14 KO mice. Quantification of immunofluorescent stainings for the pan-inflammatory cell marker CD45 (**A**) and the pan-macrophage marker F4/80 (**B**) reveal that CDE-fed Fn14 KO mice have significantly reduced pancreatic inflammation compared with the chronically injured WT controls. (**A**) Data are presented as means ± SEM, with statistical significance represented as * *p* < 0.05 and ** *p* < 0.01 comparing groups of the same genetic background and # *p* < 0.05 comparing CDE-treated WT and Fn14 KO mice. (**B**) Data are presented as means ± SEM, with statistical significance represented as *** *p* < 0.001 comparing groups of the same genetic background and ### *p* < 0.001 comparing CDE-treated WT and Fn14 KO mice.

**Figure 7:**
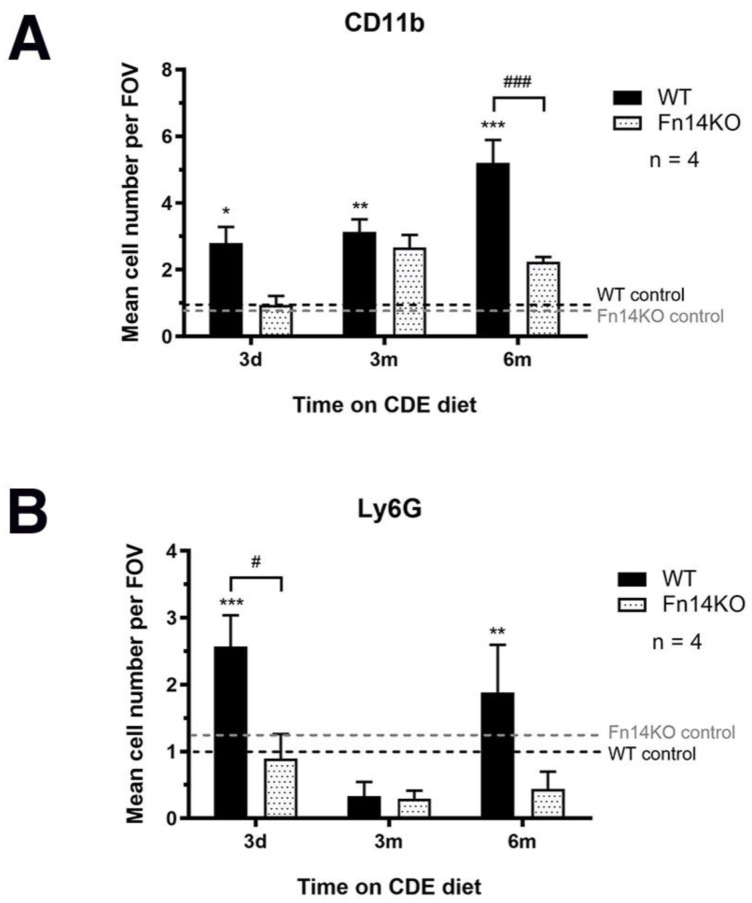
Myeloid and neutrophil cell numbers are significantly reduced in pancreata of CDE-treated Fn14 KO mice. Quantification of immunofluorescent stainings for CD11b^+^ myeloid infiltrates (**A**) and Ly6G^+^ neutrophils (**B**) demonstrate a significant reduction in cell numbers of both populations in CDE-treated Fn14 KO mice compared with chronically injured WT controls (photomicrographs shown in Supplementary Figures S3 and S4). (**A**) Data are presented as means ± SEM, with statistical significance represented as * *p* < 0.05, ** *p* < 0.01 and *** *p* < 0.001 comparing groups of the same genetic background and ### *p* < 0.001 comparing CDE-treated WT and Fn14 KO mice. (**B**) Data are presented as means ± SEM, with statistical significance represented as ** *p* < 0.01 and *** *p* < 0.001 comparing groups of the same genetic background and # *p* < 0.05 comparing CDE-treated WT and Fn14 KO mice.

As both cellular compartments, pancreatic ductular and stellate cells, respond to inflammatory signals in their microenvironment, we next evaluated all of the experimental groups for CD45 (general inflammatory cell marker) and F4/80 (pan-macrophage marker) expression. In all of our experiments, we included an early time point at day three of CDE treatment to facilitate the analysis of the direct effects of signalling ablation before other pathways may compensate. This was particularly important for the investigation of distinct inflammatory cell populations. Wildtype mice showed a quick upregulation of CD45^+^ inflammatory cells with CDE-induced pancreatic injury. In contrast, Fn14 knockout mice were delayed in their inflammatory response and still showed significantly lower CD45^+^ cell numbers at the six-month time point (Figure 6A). We next examined F4/80^+^ macrophage cell numbers, which were similar for mice of both genotypes in the acute phase of injury, suggesting that tissue-resident macrophages, which do not rely on recruitment through the TWEAK/Fn14 signalling pathway, may be the first responders. However, with chronic pancreatic injury, Fn14 knockout mice displayed significantly lower numbers of F4/80^+^ cells compared with their CDE-fed wildtype counterparts (Figure 6B). To further discern specific macrophage lineages, we then evaluated the presence of CD11b^+^ myeloid infiltrates and the numbers of Ly6G^+^ neutrophils. Both cell populations were significantly reduced in CDE-treated Fn14 knockout compared with wildtype mice in the acute three-day phase (Figure 7A,B), explaining the reduction in CD45^+^ inflammatory cell numbers (Figure 5A), most likely resulting from the lack of chemoattractant Fn14 downstream signalling. While CD11^+^ monocyte-derived macrophage numbers increased slightly over time, they were still at significantly lower numbers at six months; however, Ly6G^+^ neutrophil numbers remained low over the entire time course in Fn14 knockout animals compared with the wildtype CDE-fed mice (Figure 7B).

## 4. Discussion

Since its discovery in 1997 by Chicheportiche and colleagues [20], the TWEAK cytokine has been known to regulate a diverse range of cellular processes, including proliferation, differentiation, inflammation, angiogenesis, cell migration, and apoptosis [21]. Its cognate receptor Fn14 was initially described as an immediate-early response gene in the mouse fibroblast line NIH3T3 [22] and was later reported to be significantly upregulated in a variety of conditions associated with inflammatory tissue remodelling, repair, and cancer, making it a promising therapeutic target [23,24]. Most research into the regulatory role of the TWEAK/Fn14 pathway in a gastrointestinal context has focussed on its involvement in chronic liver disease. While the pathway activity is minimal in healthy liver tissue, Fn14 expression levels increase significantly and mediate the response to injury, as observed in an acute model using partial hepatectomy [25], as well as numerous chronic liver injury models [8,10,26,27,28,29]. In all these studies, the Fn14 downstream activity was shown to regulate inflammation in response to epithelial cell damage, proliferation of liver progenitor or ductal cells, and fibrosis-associated matrix remodelling via the activity of hepatic stellate cells.

As the liver and the pancreas both share a common embryological origin with specification from adjacent domains of the ventral foregut endoderm, it is not surprising that both organs share similar mechanisms during chronic injury [30,31]. Consistent with pathological processes in liver injury, chronic pancreatitis, as a potential precursor to pancreatic ductal adenocarcinoma, is also characterised by epithelial cell damage, leading to inflammation and ductal proliferation, and the activation of pancreatic myofibroblasts that mediate fibrotic tissue changes to provide architectural support [19,32,33,34]. Interestingly, the potential role of the TWEAK/Fn14 pathway in pancreatitis has never been investigated and, thus, became the focus of the current study.

Using The Human Cancer Genome Atlas data and other publicly available datasets, we confirmed that a high expression of Fn14 was observed in pancreatic adenocarcinoma compared with non-tumorous tissue and was associated with reduced patient survival. In addition, Fn14 was significantly upregulated in acute and chronic pancreatitis, with ductal cells representing the main Fn14^+^ pancreatic cell population, similar to Fn14-expressing liver progenitor and reactive biliary cells during chronic liver injury [8,10,27]. Before any mechanistic investigations into TWEAK/Fn14 activity could be undertaken, it was first necessary to validate the CDE diet as an appropriate model to study chronic pancreatitis. A histopathological analysis using the pancreata of mice subjected to CDE treatment for six months compared with healthy age-matched mice demonstrated hallmarks of chronic pancreatitis, including peri-acinar inflammation, proliferation of dilated interlobular ducts, and significantly reduced zymogenic granules and amylase expression, consistent with human exocrine pancreatic insufficiency. The histopathology of CDE-treated Fn14 KO mice, however, showed significantly less severe tissue changes, suggesting a disease-progressing role of active TWEAK/Fn14 signalling in chronic pancreatitis. Importantly, an immunofluorescent analysis of pancreatic islet cell populations in CDE-treated mice revealed that the endocrine pancreas was not affected. Thus, further investigations into the role of the TWEAK/Fn14 pathway in chronic pancreatitis focused on exocrine and epithelial pancreatic injury responses in the context of chronic CDE exposure are necessary.

As it is well-established that pro-inflammatory stimuli during chronic pancreatitis can lead to ductal hyperplasia and induce acinar-to-ductal metaplasia, which is strongly associated with progression to pancreatic adenocarcinoma [35], we investigated the expression of the ductal markers CK7 and CK19 in healthy and CDE-treated wildtype and Fn14 KO mice. While CK7- and CK19-positivity was restricted to normal ductal cells in the healthy pancreas, the number of both CK7^+^ and CK19^+^ cells significantly increased during long-term CDE treatment, with a significant delay in respective cell proliferation in mice that lacked Fn14 downstream signalling. The difference between CDE-treated Fn14 WT and Fn14 KO mice was particularly pronounced at the 3-month time point, where the CK19^+^ ductal cell population was represented at significantly lower levels in the Fn14 KO animals compared with the wildtype pancreatitis controls. Both markers are strongly associated with tumorigenesis, with the vast majority of pancreatic adenocarcinomas strongly staining for CK7 and CK19 [36,37]. Thus, a significant reduction in ductal proliferation in CDE-fed mice lacking Fn14 activity represents therapeutically promising data for potentially targeting this pathway in chronic pancreatitis to inhibit the progression to cancer. However, pancreatitis-to-adenocarcinoma conversion is highly multi-factorial. We, therefore, also investigated other hallmarks of chronic pancreatitis that represent known risk factors—pancreatic fibrosis and inflammation. Consistent with previous studies in chronic liver disease [8,9,10,26], a lack of Fn14 downstream signalling was (a) fibro-protective during disease progression, with significant reductions in collagen deposition at later time points and (b) resulted in significantly reduced numbers of CD45^+^ inflammatory cells, as a result of a delayed inflammatory response. Further detailed investigation of inflammatory cell types revealed that F4/80^+^ macrophages, and more specifically the CD11b^+^ cell population, as well as Ly6G^+^ neutrophils, were strongly affected by a lack of Fn14 signalling activity.

Studies of TWEAK/Fn14 signalling in pancreatic homeostasis and disease are in their early stages and it is currently not clear whether the different pathobiological responses observed in CDE-treated Fn14 knockout pancreata are regulated through direct or indirect effects of a lack of TWEAK signalling. Many studies have assessed the roles of this TNF family pathway in a liver context and revealed that ductular cells and hepatic stellate cells are the main Fn14-producing cells, which then crosstalk to other surrounding cells including resident and recruited inflammatory cell populations [9]. Our bioinformatic analysis of publicly available datasets proposes ductular cells as the main Fn14^+^ population. We therefore hypothesise that similar dynamic mechanisms are at play during chronic pancreatitis to regulate TWEAK/Fn14-dependent fibrogenesis- and inflammation-associated downstream effects. 

Regardless of the precise signalling networks orchestrating the observed biological responses, these data demonstrate that therapeutic targeting of TWEAK/Fn14 signalling is not only a promising avenue to potentially inhibit liver disease progression, but may be equally warranted in the chronic pancreatitis setting.

Previous studies have reported significant anti-tumoral effects when TNF itself was targeted in pancreatic end-stage disease. Egberts and colleagues used a preclinical pancreatic ductal adenocarcinoma model and showed a significant reduction in tumour growth and metastasis when TNF was inhibited by infliximab or etanercept treatment [38]. More recently, anti-TNF therapy by infliximab or etanercept treatment in combination with gemcitabine or paclitaxel chemotherapy was investigated using an orthotopic pancreatic cancer patient-derived xenograft mouse model and was found to be effective in delaying tumour growth and improving survival [39]. As TWEAK is a TNF family member, targeting the TWEAK ligand, its receptor Fn14, or the induced downstream signalling seems to be an obvious next therapeutic avenue that should be explored. This study will now serve as a fundamental basis for further mechanistic investigations into the role of the TWEAK/Fn14 pathway, not only in established pancreatic disease, but also to therapeutically target Fn14 downstream effects for regulating the tissue microenvironment in chronic pancreatitis.

## 5. Conclusions

This study confirms CDE feeding as a suitable model to study the key features of chronic pancreatitis, and is the first report to highlight important regulatory roles of TWEAK/Fn14 signalling in chronic pancreatic disease establishment and progression.

## Data Availability

The data presented in this study are available upon request from the corresponding author.

## Supplementary Materials

The following supporting information can be downloaded at: https://www.mdpi.com/article/10.3390/cancers15061807/s1. Supplementary Figure S1: Lack of Fn14 signalling activity delays the pancreatic ductular reaction. Immunofluorescent staining and quantitation of cytokeratin (CK) 19+ pancreatic ducts revealed a delay in ductular proliferation in CDE-fed Fn14 KO compared to chronically injured WT mice. Supplementary Figure S2: Lack of Fn14 signalling activity significantly reduces CDE-induced fibrosis development. Immunohistochemical staining for a-smooth muscle actin (a-SMA)+ pancreatic stellate cells demonstrated significantly reduced fibrogenesis in CDE-fed Fn14 KO compared to chronically injured WT mice. Supplementary Figure S3: Myeloid cell numbers are significantly reduced in pancreata of CDE-treated Fn14 KO mice. Quantification of immunofluorescent stainings for CD11b+ myeloid infiltrates demonstrated a significant reduction in cell numbers in CDE-treated Fn14 KO mice compared to chronically injured WT controls. Supplementary Figure S4: Neutrophil cell numbers are significantly reduced in pancreata of CDE-treated Fn14 KO mice. Quantification of immunofluorescent stainings for Ly6G+ neutrophils demonstrated a significant reduction in cell numbers in CDE-treated Fn14 KO mice compared to chronically injured WT controls.
