# Supplementary figures and images for: TWEAK/Fn14 Signalling Regulates the Tissue Microenvironment in Chronic Pancreatitis
Source: Cancers (Basel). 2023 Mar 16;15(6):1807. doi: 10.3390/cancers15061807 (PMC10046490; doi:10.3390/cancers15061807)

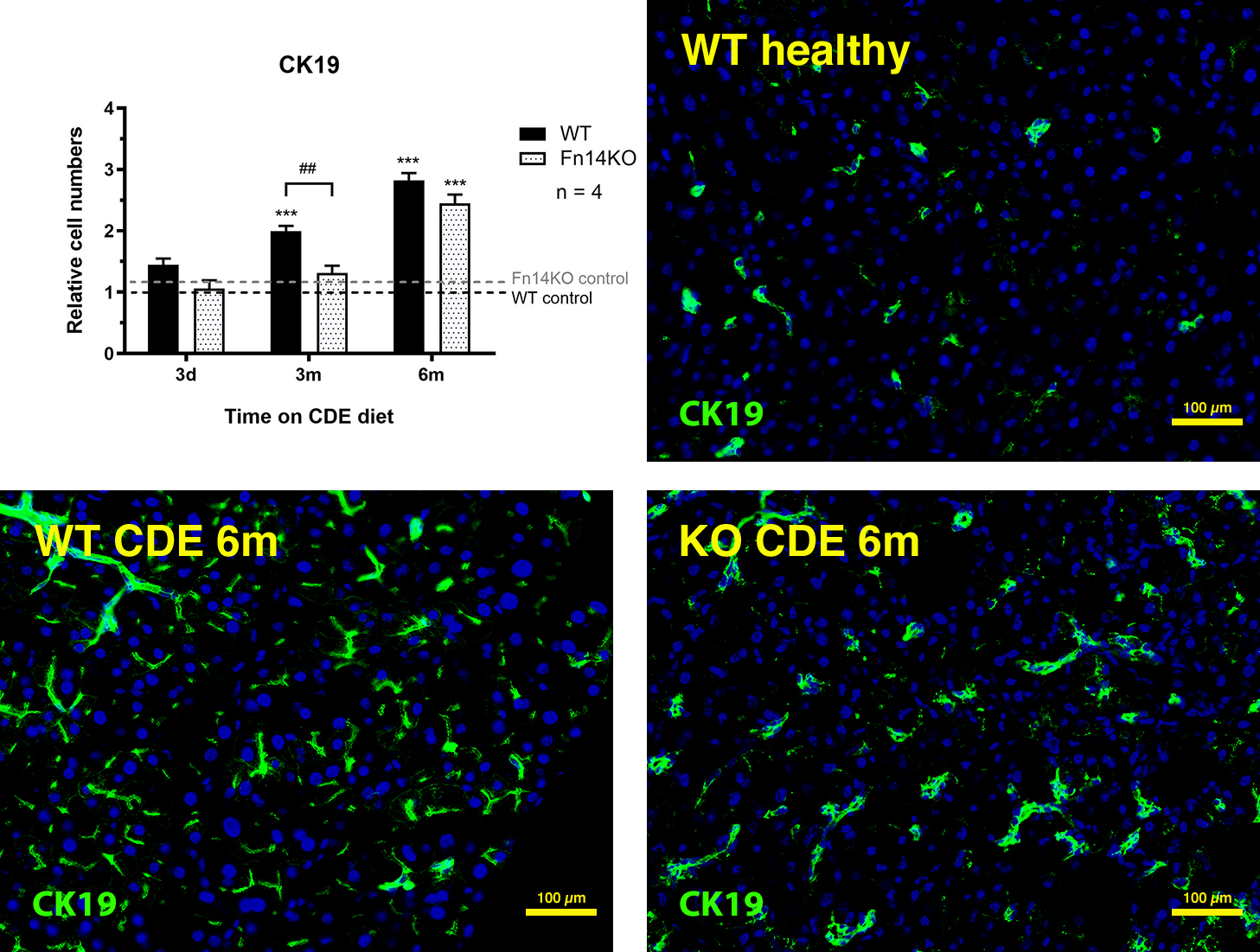

Supplement: Supplementary file 1 [file cancers-15-01807-s001.zip › Supplementary Figure S1 - CK19.jpg]

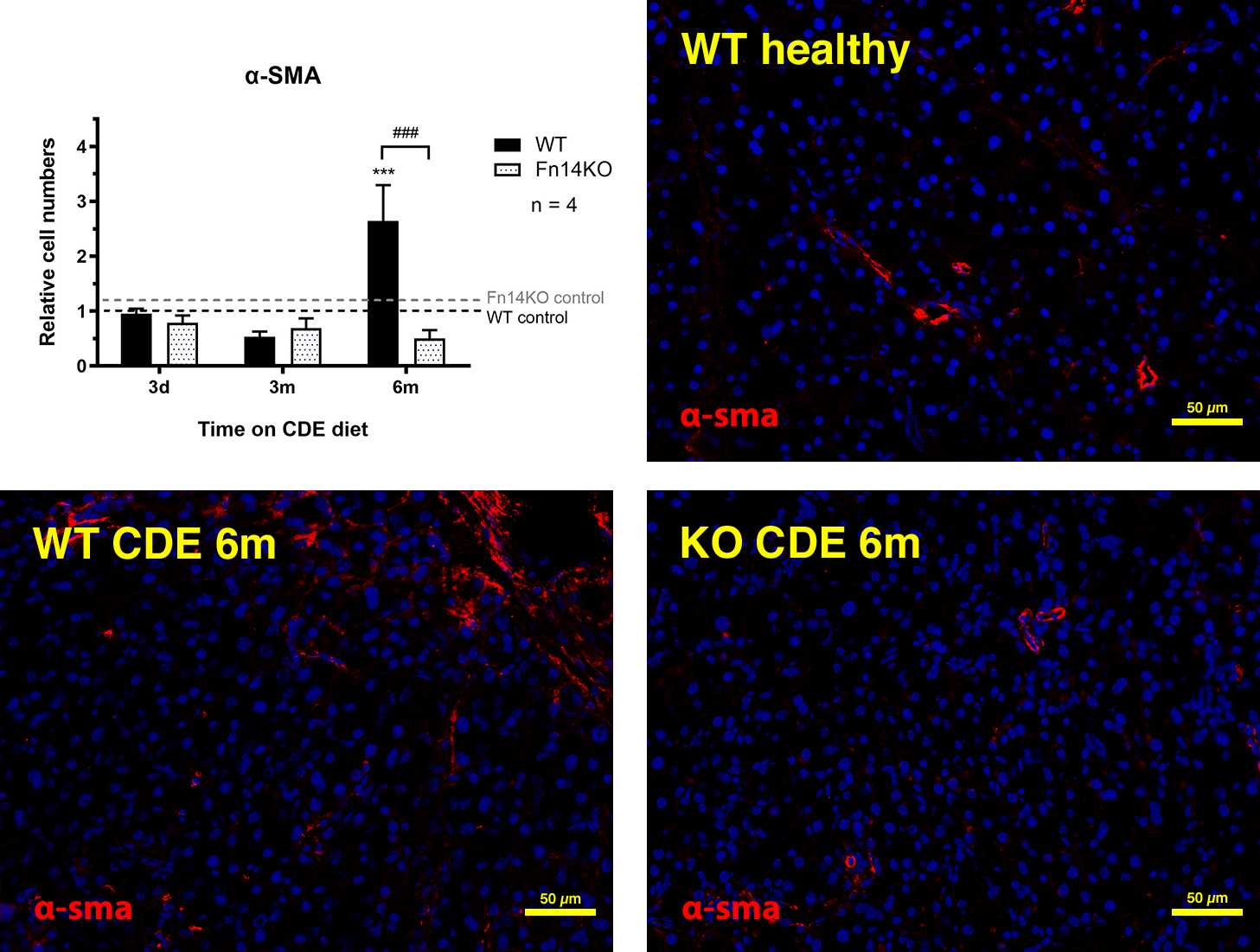

Supplement: Supplementary file 1 [file cancers-15-01807-s001.zip › Supplementary Figure S2 - aSMA.jpg]

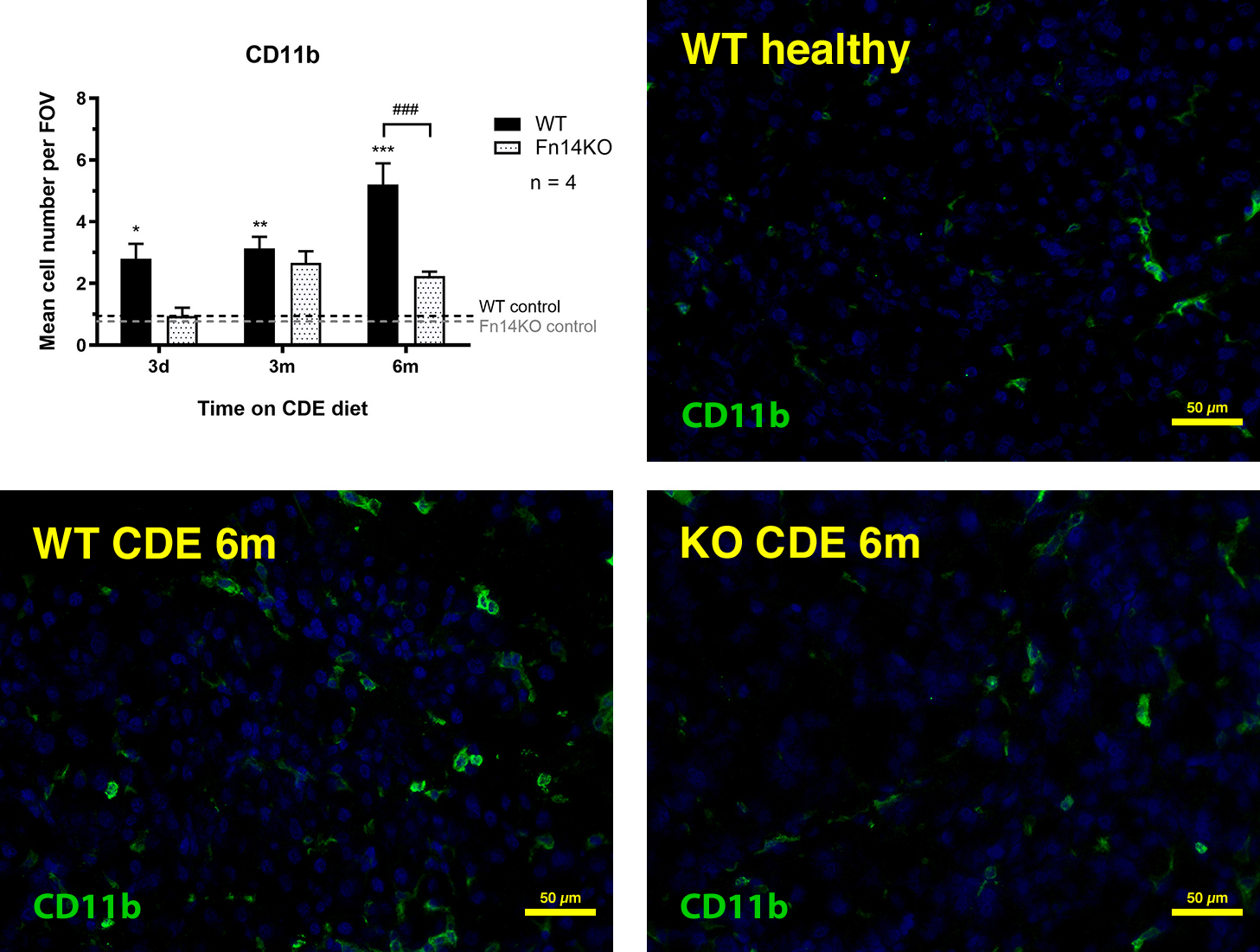

Supplement: Supplementary file 1 [file cancers-15-01807-s001.zip › Supplementary Figure S3 - CD11b.jpg]

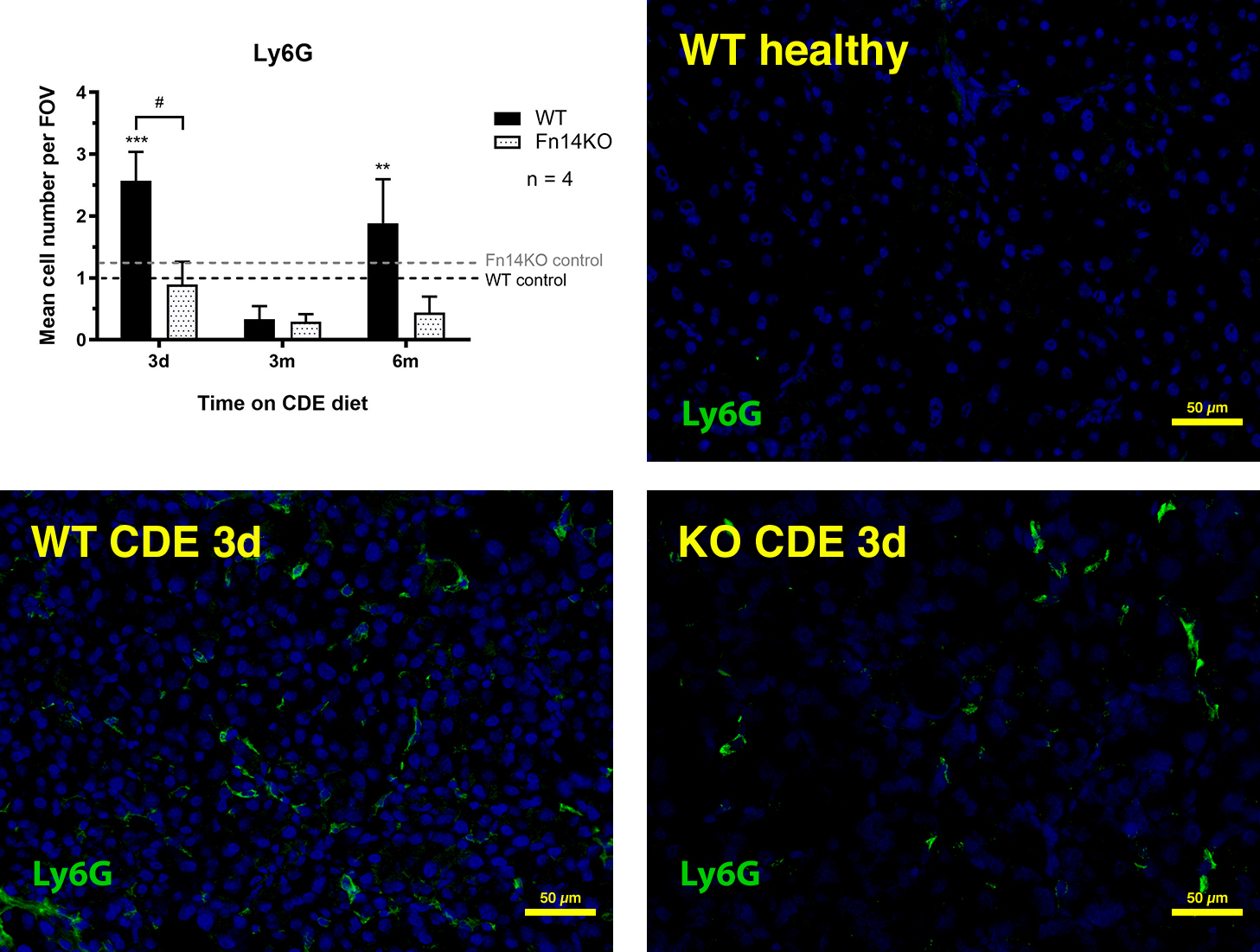

Supplement: Supplementary file 1 [file cancers-15-01807-s001.zip › Supplementary Figure S4 - Ly6G.jpg]
